# Effects of Light on the Fruiting Body Color and Differentially Expressed Genes in *Flammulina velutipes*

**DOI:** 10.3390/jof10060372

**Published:** 2024-05-22

**Authors:** Ji-Hoon Im, Che-Hwon Park, Ju-Hyeon Shin, Youn-Lee Oh, Minji Oh, Nam-Chon Paek, Young-Jin Park

**Affiliations:** 1 Department of Agriculture, Forestry and Bioresources, College of Agriculture and Life Sciences, Seoul National University, Seoul 08826, Republic of Korea; jihooni24@korea.kr; 2 Mushroom Research Division, National Institute of Horticultural and Herbal Science, Rural Development Administration, Eumseong-gun 27709, Republic of Korea; o5ne2@korea.kr (Y.-L.O.); minji1228@korea.kr (M.O.); 3 Department of Medicinal Biosciences, Research Institute for Biomedicinal & Health Science, College of Biomedicinal and Health Science, Konkuk University, Chungju 27478, Republic of Korea; chehwon9798@kku.ac.kr (C.-H.P.); shin99@kku.ac.kr (J.-H.S.)

**Keywords:** *Flammulina velutipes*, light, fruiting body color, transcriptome, cytochrome P450, pyridoxal-dependent decarboxylases

## Abstract

Light plays vital roles in fungal growth, development, reproduction, and pigmentation. In *Flammulina velutipes*, the color of the fruiting body exhibits distinct changes in response to light; however, the underlying molecular mechanisms remain unknown. Therefore, in this study, we aimed to analyze the *F. velutipes* transcriptome under red, green, and blue light-emitting diode (LED) lights to identify the key genes affecting the light response and fruiting body color in this fungus. Additionally, we conducted protein–protein interaction (PPI) network analysis of the previously reported fruiting body color-related gene, *Fvpal1*, to identify the hub genes. Phenotypic analysis revealed that fruiting bodies exposed to green and blue lights were darker than those untreated or exposed to red light, with the color intensifying more after 48 h of exposure to blue light compared to that after 24 h of exposure. Differentially expressed gene (DEG) analyses of all light treatments for 24 h revealed that the numbers of DEGs were 17, 74, and 257 under red, green, and blue lights, respectively. Subsequently, functional enrichment analysis was conducted of the DEGs identified under green and blue lights, which influenced the color of *F. velutipes*. In total, 103 of 168 downregulated DEGs under blue and green lights were included in the enrichment analysis. Among the DEGs enriched under both green and blue light treatments, four genes were related to monooxygenases, with three genes annotated as cytochrome P450s that are crucial for various metabolic processes in fungi. PPI network analysis of Fvpal1 revealed associations with 11 genes, among which the expression of one gene, pyridoxal-dependent decarboxylase, was upregulated in *F. velutipes* exposed to blue light. These findings contribute to our understanding of the molecular mechanisms involved in the fruiting body color changes in response to light and offer potential molecular markers for further exploration of light-mediated regulatory pathways.

## 1. Introduction

The edible fungus *Flammulina velutipes* exhibits morphological diversity with taxonomic classification under division Basidiomycota, class Agaricomycetes, order Agaricales, family *Physalacriaceae*, and genus *Flammulina*. Although its commercial varieties typically exhibit white coloration, wild species with yellow-to-dark brown coloration are commonly found in nature. Moreover, commercial varieties have slender and elongated stipes and small pilei, whereas wild species exhibit various shapes and sizes for both stipes and pilei, with considerable morphological variation [1]. These mushrooms are commonly found on decaying old trees or remnants of various broadleaf trees from late autumn until the subsequent spring. This particular mushroom species exhibits a remarkable ability to withstand cold temperatures, allowing it to flourish even during the harsh winter months, and thus it is also known as “Winter mushroom.” The resilience displayed by *F. velutipes* in surviving adverse environmental conditions further highlights its adaptive nature and ecological significance in its natural habitat.

Fungi, as dependent heterotrophs, have evolved morphologically to respond to various environmental factors, such as soil composition and climate; generate fruiting bodies of mushrooms; and effectively disperse spores at the most suitable locations and times. Environmental factors that influence the form and coloration of mushrooms include temperature, humidity, light, and CO_2_ levels. Light, in particular, is a crucial determinant that influences various facets of fungal biology, including the growth and development of mycelia and fruiting bodies [2,3,4]. The mycelial growth of *Auricularia heimuer* was strong under a red light to blue light ratio of 1:1 [5], but the mycelia of most fungi thrive under low light or even completely dark conditions. In *Lentinula edodes*, the cultivation medium did not produce a brown film under 80 days of darkness, whereas it did form a brown film after 30 days of darkness followed by 50 days of light exposure [6]. Blue light significantly promoted the growth and development of the stipe, pileus, and gill of *Pleurotus ostreatus*, especially the pileus, while red light had a minor inhibitory effect on pileus growth [7]. In addition, the growth response of *Hypsizygus marmoreus* varied depending on the light treatment during cultivation, primordia formation, and growth stages [8]. It also enhances fruiting body uniformity, yield, and biological efficiency in *F. filiformis* and increases the primordia and fruiting body yields in *H. marmoreus* [8,9].

These photomorphogenesis phenomena, wherein light serves as a key environmental cue that regulates growth and development, affect mushroom pigmentation. Understanding the intricate interplay between light exposure and mushroom pigmentation is necessary to explore their ecology, physiology, and potential applications [10,11,12]. Light intensity, quality, duration, and directionality strongly influence the expression of genes involved in pigment biosynthesis pathways [7,13,14]. For example, ultraviolet (UV) light exposure stimulates the production of melanin-like pigments in some mushroom species as a protective response to UV radiation [15,16]. Spectral composition of light modulates the synthesis of specific pigments in mushrooms. Different wavelengths of light, ranging from blue to red, elicit differential responses for metabolite production and accumulation [14,17,18]. For example, in *P. ostreatus*, blue light leads to an accumulation of the aromatic amino acid shikimic acid due to the increased activity of 3-deoxy-D-arabinoheptulosonate 7-phosphate synthase (DAHPS), phosphofructokinase (PFK), and glucose-6-phosphate dehydrogenase (G6PD) [17]. In *L. edodes*, blue light induces changes in the expression of genes related to morphological development and pigment production in fruiting bodies [13]. Additionally, in *Terana caerulea*, exposure to blue light triggers the production of inkblue pentacyclic natural products, known as corticin pigments [14]. Therefore, influence of light on mushroom pigmentation extends beyond coloration, encompassing ecological interactions and adaptive strategies. In natural habitats, mushrooms exhibit phenotypic plasticity in response to varying light conditions, with coloration patterns tailored to optimize their survival and fitness [19]. Mushrooms inhabiting shaded environments exhibit lighter pigmentation to maximize light absorption, whereas those exposed to direct sunlight exhibit darker pigmentation for UV protection and heat dissipation. Moreover, effects of environmental factors on mushroom pigmentation extend beyond natural ecosystems to include various agricultural and industrial applications [11].

Advancements in genomic, transcriptomic, and proteomic technologies have facilitated elucidation of the molecular basis of light-induced pigmentation in mushrooms. High-throughput sequencing, gene expression profiling, and functional analyses have enabled the identification and characterization of key genes and regulatory elements governing pigment biosynthesis pathways [7,12,13,14,20,21]. For example, in *P. ostreatus*, blue light increased the activation of glycolysis and the pentose phosphate pathway, including enzymes encoding genes like 6-phosphogluconate dehydrogenase (6PGD) and phosphoenolpyruvate carboxykinase (PEPCK) [7]. In *Terana caerulea*, exposure to blue light resulted in significantly higher transcription of the gene encoding the gateway enzyme, polyporic acid synthetase CorA, which catalyzes the formation of the pigment core structure [14]. In *F. velutipes*, the gene *FvbHLH1* exhibited unique expression specifically in the yellow cap, suggesting its potential regulatory role in phenolic acid biosynthesis [22]. And isobavachalcone D, a yellow compound, and the control of riboflavin transportation by MCH5 play crucial roles in the development of the yellow cap in *P. citrinopileatus* [23]. Previous studies have explored mushroom responses to light and investigated color-related aspects; however, the molecular mechanisms underlying the relationship between light and mycelial color remain unclear. As the light response mechanisms of mushrooms vary among species [24], the functional significance of these mechanisms needs to be assessed in different conditions. Therefore, in this study, we performed transcriptome analysis of *F. velutipes* irradiated using red, green, and blue light-emitting diodes (LEDs) to identify the key genes associated with light response and fruiting body color. Additionally, we conducted protein–protein interaction (PPI) network analysis of a previously identified fruiting body color-related gene, *Fvpal1* [25], to identify the hub genes. The light-responsive and fruiting body color-related genes identified in this study provide new insights into the light response mechanisms of fungi and may be used as selective markers for color changes in *F. velutipes*.

## 2. Materials and Methods

### 2.1. Fungal Strains, Fruiting Body Cultivation, and Light Treatment

*F. velutipes* strain ASI 4232 was obtained from the Mushroom Division of the Rural Development Administration (Eumsung-gun, Republic of Korea) and cultured on potato dextrose agar (Difco, Seoul, Republic of Korea) at 25 °C for 20 d. Then, *F. velutipes* mycelia were inoculated into a fruiting medium (80% sawdust and 20% rice bran) and incubated at 18 °C under 65% humidity for 35 d. After incubation, the mycelia were scraped from the surface of the bottles, and primordia formed at 14 °C under 90–95% humidity were grown at 6–7 °C under 80–85% humidity for fruiting. They were exposed to red (639 nm), green (522 nm), and blue (470 nm) LEDs at a distance of 20 cm with the same light intensity of 500 lx for 24 and 48 h immediately before harvesting. Each treatment was applied to four bottles containing 350 g of fruiting bodies per bottle. Colorimetric analysis was conducted using a compact portable colorimeter, the Minolta CR-400 (Konica Minolta, Osaka, Japan), for all bottles to quantify the color of the samples.

### 2.2. Transcriptome Analysis and Differentially Expressed Gene (DEG) Identification

For transcriptome analysis, fruiting bodies harvested from three bottles per each of the four light treatments were ground in liquid nitrogen. Total RNA was isolated using the TRIzol reagent (Thermo Fisher Scientific Korea, Seoul, Republic of Korea) and purified using the RNeasy kit (Qiagen Korea, Seoul, Republic of Korea) with RNase-free DNase (Qiagen Korea), according to the manufacturers’ instructions. Purified RNA (1 μg) was sequenced on the HiSeq 2000 platform (Illumina Korea, Seoul, Republic of Korea). Quality-trimmed short reads (Phred quality score: 30) were processed using the Trinity pipeline (version 2.15.0) [26]. Additionally, TransDecoder (version 5.5.0) [27] was used for gene modeling, CD-HIT (version 4.8.1) [28] for clustering and deduplication, and DESeq2 (in Trinity pipeline) [26] for the identification of DEGs (|log2FC| ≥ 1 and *p* < 0.001). All next-generation sequencing reads have been deposited into the National Center for Biotechnology Information Sequence Read Archive (SRA) (PRJNA1107629).

### 2.3. Functional Enrichment and PPI Network Analysis of DEGs

All coding sequences (CDSs) predicted via Trinity analysis were annotated using the protein families (Pfam) [29], InterPro [30], and Kyoto Encyclopedia of Genes and Genomes (KEGG) [31] databases. The criterion for statistical significance was set as *p* < 0.001 for all database searches. The predicted CDSs and DEGs were used for functional enrichment and PPI analyses using the Search Tool for the Retrieval of Interacting Genes/Proteins (STRING) database [32]. PPI hub genes were identified using the cytoHubba application in Cytoscape software (version 3.10.1) [33].

### 2.4. Quantatitive Polymerase Chain Reaction (PCR) of DEGs

Next, qPCR was performed to evaluate the expression levels of DEGs. cDNA was synthesized by genomic DNA elimination and reverse-transcription reaction using the QuantiTect-Reverse Transcription kit (Qiagen) according to the manufacturer’s protocol. After genomic DNA elimination reaction with gDNA wipeout buffer at 42 °C for 2 min, the first-strand synthesis was conducted at 42 °C for 15 min, followed by incubation at 95 °C for 5 min to inactivate the reverse transcriptase. The synthesized cDNA (200 ng) and 1 μM of each primer (Table S1) were mixed with the QuantiTect SYBR green PCR kit (Qiagen) and amplified using the Rotor-Gene Q instrument (Qiagen). Relative quantification of gene expression was performed using the ^ΔΔ^Ct method.

## 3. Results

### 3.1. Effect of Light on the Fruitng Body Color

**Figure 1:**
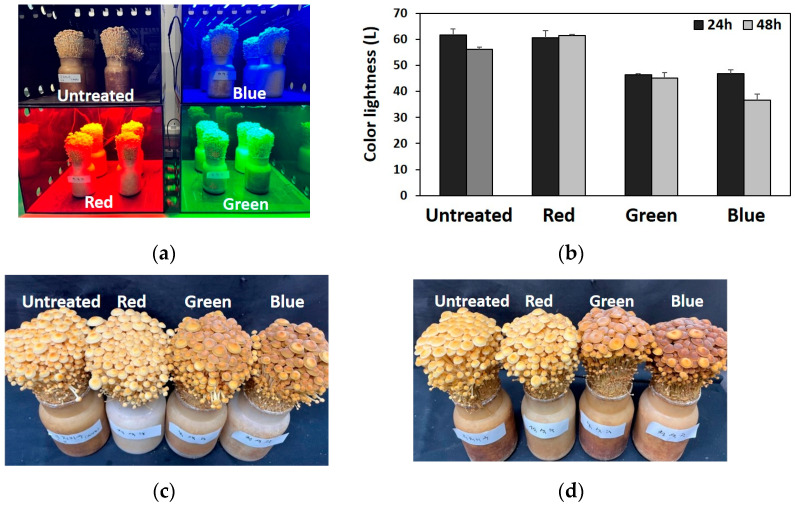
Light treatment of *Flammulina velutipes* fruiting bodies. (**a**) Light-emitting diode (LED) treatment. (**b**) Colors of the light-treated fruiting bodies (**c**) 24 h and (**d**) 48 h after light treatment.

Here, three different LED lights (red, green, and blue) were used to irradiate the *F. velutipes* fruiting bodies immediately before harvest. As shown in Figure 1, colors of fruiting bodies exposed to green and blue lights were darker than those of the unexposed and red light-exposed fruiting bodies. Moreover, under blue light treatment, color of the fruiting body was darker after 48 h than after 24 h (Figure 1b). Notably, red light treatment did not affect the fruiting body color, which was similar to the color of the untreated fruiting bodies (Figure 1c,d). After determining the light treatment affected the fruiting body color, we performed transcriptome analysis to identify the specific genes involved in the response to light treatment.

### 3.2. DEG Identification

**Figure 2:**
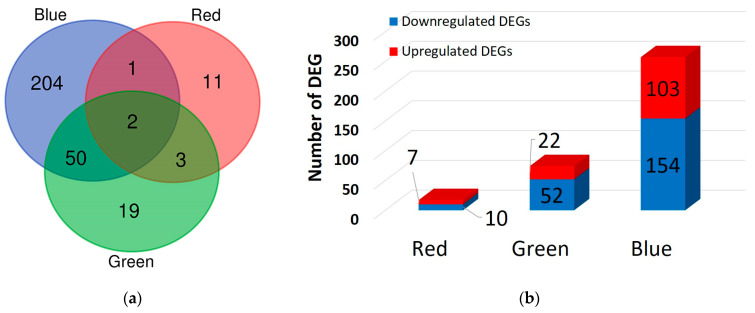
Venn diagram (**a**) and number (**b**) of differentially expressed genes (DEGs) identified after the red, green, and blue light treatments of *F. velutipes*.

Next, DEGs were identified to assess the response of genes to light treatment as well as the resulting changes in the fruiting body color of *F. velutipes*. Figure 2 shows the numbers of DEGs after red, green, and blue light treatments compared to those in untreated *F. velutipes* using |log2FC| ≥ 1 and *p* < 0.05 as the threshold (Table S2). DESeq2 analysis revealed 17, 74, and 257 DEGs in the red-, green-, and blue-light-treated *F. velutipes*, respectively. Specifically, two DEGs (TRINITY_DN3502_c0_g1_i6 and TRINITY_DN363_c0_g1_i12) were detected in all light treatments, and their levels were upregulated in red-, green-, and blue-light-treated *F. velutipes* (Figure 2; Table S2). Of these, TRINITY_DN3502_c0_g1_i6 was annotated as a fungus-specific transcription factor (TF) domain (PF04082.22) via the Pfam database search. As shown in Figure 2b, the highest number of DEGs was observed in blue-light-treated *F. velutipes*. A relatively small number of DEGs was identified in red-light-treated *F. velutipes*.

### 3.3. Functional Enrichment of DEGs

**Figure 3:**
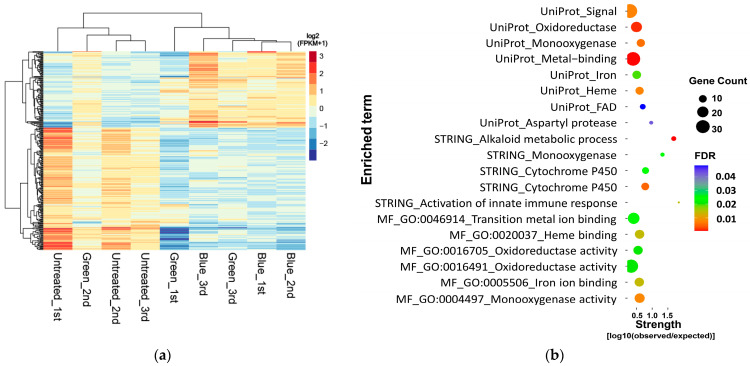
Heat map (**a**) and significantly enriched terms (**b**) of 168 downregulated DEGs in blue- and green-light-treated *F. velutipes*.

In total, 290 genes were identified as DEGs in light-treated *F. velutipes*, and 273 DEGs were identified in samples treated with only blue and green lights, but not in those treated with red light. Figure 3a shows a heatmap of the 105 upregulated and 168 downregulated genes among the identified 273 DEGs. Then, DEG enrichment analysis was conducted using the STRING database. Among the 273 DEGs, only 168 downregulated DEGs in blue- and green-light-treated *F. velutipes* were enriched and classified using the Gene Ontology (GO) molecular function (MF), STRING cluster, and UniProt keywords (Figure 3b; Table S3). These downregulated DEGs were significantly enriched in three categories of the STRING database: monooxygenase activity (GO:0004497) in MF, alkaloid metabolic process in the STRING cluster, and monooxygenase in UniProt keywords. Enrichment analysis revealed that the downregulated DEGs were enriched in monooxygenase activity in all three categories (Table S3).

A total of 103 of the 168 downregulated DEGs in blue- and green-light-treated *F. velutipes* were included in the enrichment analysis (Table S4). Among these, 30, 31, and 79 genes were annotated using the KEGG, InterPro, and Pfam databases, respectively. In addition, 4 out of 16 monooxygenase-related genes were commonly identified as enriched DEGs in both blue- and green-light-treated *F. velutipes* (Table 1 and Table S4). Among these four genes, three were annotated as cytochrome P450 (CYP) enzymes using the Pfam database.

### 3.4. Expression Patterns and PPI Networks of the F. velutipes Fruiting Body Color-Related Genes

**Figure 4:**
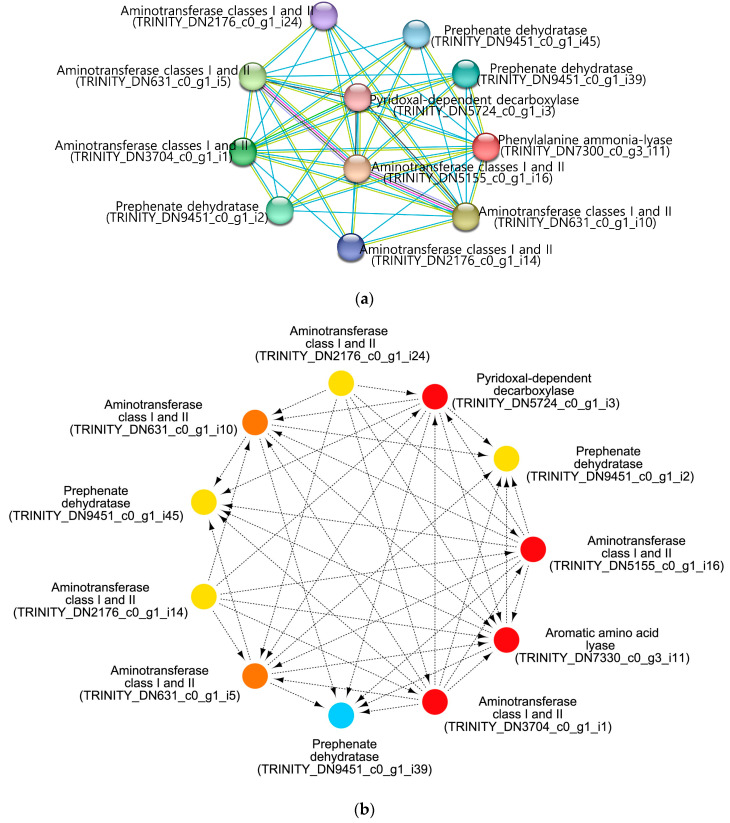
PPI network analysis of Fvpal1 using STRING DB (**a**) and identification of hub genes associated with *Fvpal1* by CytoHubba application; arrows indicate interactions (**b**).

We previously identified a gene related to the fruiting body color of *F. velutipes* [25]. Comparative genomic analysis of different *F. velutipes* strains revealed 70 white strain-specific variations, including single-nucleotide polymorphisms and indels, with one mutation causing a deletion in the phenylalanine ammonia-lyase 1 (*Fvpal1*; EC 4.3.1.24) gene. This mutation is color-specific for white strains and plays an important role in determining the color of the fruiting body. Here, *Fvpal1* was identified as an upregulated DEG (log2FC 1.27) only in blue-light-treated *F. velutipes* (Table S2). Further analysis, including enrichment and PPI network analyses of *Fvpal1* (TRINI-TY_DN7330_c0_g3_i11), was performed using the STRING database and Cytoscape software. As shown in Figure 4a, 11 nodes and 44 edges were obtained with scores > 0.4. CytoHubba application of Cytoscape software was used to identify the hub genes in the PPI network (Figure 4b). All identified hub genes with closeness and degree of connectivity are listed in Table 2 and Table S5. Among the identified genes, only one gene (TRINITY_DN5724_c0_g1_i3) acted as an upregulated DEG in blue-light-treated *F. velutipes*. This gene was annotated to have a pyridoxal-dependent decarboxylase-conserved domain using the Pfam database.

### 3.5. qPCR Validation of DEGs

**Figure 5:**
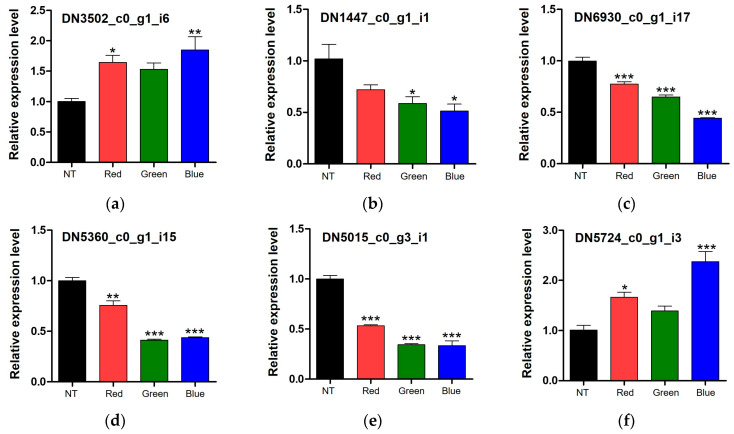
Relative expression levels of the upregulated genes under all light conditions (**a**), downregulated genes under blue and green lights (**b**–**e**), and upregulated genes under blue light (**f**). Data were analyzed using one-way analysis of variance (ANOVA) followed by Tukey’s test. * *p* < 0.05, ** *p* < 0.01, and *** *p* < 0.001 vs. non-treated sample. NT, non-treated; Red, red LED treated; Green, green LED treated; Blue, blue LED treated.

The DEGs identified from the fruiting bodies of *F. velutipes* exposed to light were analyzed using quantitative reverse transcription-PCR (qRT-PCR). One upregulated gene (TRINITY_DN3502_c0_g1_i6) under all light conditions (Figure 5a), four downregulated genes (TRINITY_DN1447_c0_g1_i1, TRINITY_DN6930_c0_g1_i7, TRINITY_DN5360_c0_g1_i15, and TRINITY_DN5015_c0_g3_i1) enriched only in blue- and green-light-treated *F. velutipes* via functional enrichment analysis (Figure 5b–e), and one upregulated gene (TRINITY_DN5724_c0_g1_i3) only under blue light (Figure 5f) via PPI network analysis of *Fvpal1* were used for validation of the transcriptome data. These genes showed similar expression patterns in qPCR and transcriptome analysis results (Figure 5). These results confirmed the reliability of transcriptome data and suggested the potential of the identified DEGs, responsive to light and associated with the color of fruiting bodies in *F. velutipes*, for further exploration of genes of interest.

## 4. Discussion

Fruiting body forming fungi exhibit a sophisticated ability to perceive various environmental cues, enabling them to meticulously assess the factors influencing the selection of ideal sites and timing of sexual reproduction. Additionally, these fungi possess a remarkable capacity to craft suitable fruiting bodies to ensure efficient dispersal of spores, thereby enhancing their reproductive success in the ecosystem. Light plays key roles in plant and fungal morphogenesis, primarily because of its significance in photosynthesis and morphogenesis. The effects of light on various biological systems have been extensively studied in ascomycetes [34,35,36]. Light perception is essential for the spatial recognition of mushroom-forming fungi, particularly in sexual reproduction. However, the correlation between light exposure and fruiting body formation in basidiomycetes remains unknown.

The necessity of light for the induction of fruiting bodies varies among basidiomycetes types. Some species can initiate fruiting body production in the absence of light, suggesting that light is not a critical factor for this process [37,38,39]. Nevertheless, light has the capability to trigger or enhance the production of fruiting bodies [40,41,42,43]. Wavelengths that effectively induce the formation of fruiting bodies include UV (280 nm) and blue (520 nm) lights [7,44,45].

Here, to examine the effects of different LED light sources on the morphological characteristics of *F. velutipes* fruiting bodies, *F. velutipes* was irradiated with blue, green, and red LED lights for 24 and 48 h after fruiting body formation. The color of the fruiting body darkened when exposed to both green and blue lights, but a more pronounced effect was noted with blue light. This suggests that different LED lights affect the fruiting body pigmentation. Subsequently, transcriptome analysis of fruiting bodies exposed to LED irradiation was conducted, focusing on the light-responsive genes. A total of 340 DEGs (|log2FC| ≥ 1 and *p* < 0.05) were identified, including 17 DEGs for red light, 74 DEGs for green light, and 257 DEGs for blue light. Two DEGs were consistently identified across all light treatments, with one of them annotated as a fungus-specific TF domain based on a Pfam search. White collar-1 in *Neurospora crassa* acts as a fungus-specific TF domain that responds to light and serves as a blue light photoreceptor for circadian clock regulation [46]. The fungal-specific TF domain that responds to light stimuli is the GAL4-like Zn2C6 DNA-binding domain [47]. This domain is a characteristic feature of a significant TF class in fungi and plays a crucial role in regulating gene expression in response to light signals. Previous studies have shown that this domain, along with other fungus-specific domains, such as the middle homology domain, is involved in controlling various biological processes, including growth, development, and secondary metabolite production in fungi [47,48,49]. The interaction of these specific domains within TFs forms the basis of gene regulatory networks that orchestrate light-mediated responses in fungi, such as *N. crassa* and *Cordyceps militaris* [48,49]. Therefore, our results suggest that one of the two DEGs may act as a TF involved in the light response in *F. velutipes*.

As the color of *F. velutipes* fruiting bodies darkened when exposed to green and blue light, we performed an enrichment analysis for DEGs whose expression changed in response to the two light treatments. Among the 273 DEGs, 168 downregulated genes were enriched in the online STRING database. Among the 273 DEGs, only 168 downregulated DEGs in the blue- and green-light-treated samples were enriched in GO MF, STRING cluster, and UniProt keywords. The downregulated DEGs were enriched in monooxygenases across all three categories. Among the 168 downregulated DEGs, three were identified as CYPs and downregulated in *F. velutipes* treated with both blue and green light. CYP enzymes are important for various fungal metabolic processes, including sterol synthesis, steroid oxidation, and xenobiotic degradation. These enzymes exhibit diverse functions, including hydroxylation, dealkylation, and ring formation, and play a key role in detoxification and ecological survival [50,51,52]. It has been reported that the expression of CYP in blue-light-irradiated *L. edodes* increases with fruiting body development and shows consistently high expression at all growth stages [13]. The role of CYPs in pigment biosynthesis in plants is well known [53,54,55], but the relationship between CYPs and fruiting body color in basidiomycetes is still unclear. The biological clock controls many metabolic processes in various organisms, including microorganisms, plants, and animals. Circadian rhythms are important for physiological phenomena related to the growth and development of higher plants [56,57,58]. Additionally, circadian regulation by CYP monooxygenases is important for the synthesis of diverse secondary metabolites, including phenylpropanoids, carotenoids, glucosinolates, and brassinosteroids, in *Arabidopsis thaliana* [59]. Although further studies are required, these results suggest that these downregulated CYPs are probably regulated in response to light as circadian reporters but are unlikely to directly affect *F. velutipes* fruiting body color.

We previously identified a phenylalanine ammonia lyase (*Fvpal1*) gene with a mutation (ΔGCGCAC) specific to white *F. velutipes* strains [25]. Phenylalanine ammonia-lyase facilitates the deamination of l-phenylalanine into trans-cinnamic acid. It is frequently found in plants and fungi and plays an important role in various metabolic pathways and biological processes [60]. Interestingly, this gene was upregulated in *F. velutipes* treated with blue light (Table S2). In addition, as shown in Figure 1d, among the samples treated with light for 48 h, the color of the fruiting bodies treated with blue light was darker than that of the fruiting bodies treated with red or green light. Additionally, PPI network construction and hub gene identification revealed that this gene was mainly associated with aminotransferase genes (Table 2). Among them, one gene (TRINITY_DN5724_c0_g1_i3) possessing a pyridoxal-dependent decarboxylase conserved domain was upregulated in blue-light-treated *F. velutipes* (Table S2). Pyridoxal-dependent decarboxylases are crucial enzymes in metabolic pathways involved in the biosynthesis of amino acids, amino-acid-derived metabolites, and amino sugars of living organisms, utilizing pyridoxal 5′-phosphate (PLP) as a coenzyme for decarboxylation reactions [61]. Previously, the catalytic activity of this enzyme has been reported to be light-sensitive [62]. In addition, pyridoxal-dependent decarboxylase conserved domain is important for the function of enzymes, such as DOPA decarboxylase (DDC), tyrosine decarboxylase (TDC), glutamate decarboxylase (GAD), and histidine decarboxylase (HDC) [63]. DDC (EC4.1.1.26) and TDC (EC4.1.1.25) produce tyramine using the amino acid l-tyrosine as a substrate. GAD (EC4.1.1.15) catalyzes the decarboxylation of Glu to gamma-aminobutyric acid. HDC (EC 4.1.1.22) catalyzes the decarboxylation of histidine to produce histamine. Tyrosine, phenylalanine, and tryptophan are three AAAs involved in protein synthesis and are substrates for the synthesis of various secondary metabolites. AAAs are substrates for numerous anabolic pathways responsible for the synthesis of pigment compounds, plant hormones, and biological macromolecules [64]. Therefore, the pyridoxal-dependent decarboxylase conserved domain of the gene (TRINITY_DN5724_c0_g1_i3) sequence, which was identified as a hub gene by PPI network analysis and was also upregulated by blue light treatment, suggests that this gene may be involved in the metabolism of AAAs and the pigment biosynthetic pathway.

This study conducted a comprehensive bioinformatics analysis of DEGs involved in the light response of *F. velutipes*. We identified seven genes closely related to the light response and fruiting body color changes of *F. velutipes*. These genes are key candidate markers for further investigation of the molecular mechanisms underlying the light response and fruiting body color changes in this mushroom.

## 5. Conclusions

Here, exposure to different LED light sources, particularly blue and green lights, influenced the pigmentation of *F. velutipes* fruiting bodies. Using transcriptome analysis, we identified a set of DEGs involved in the light response of this mushroom. Among the identified 168 downregulated genes, three acted as CYP genes that are important for various metabolic processes in fungi. Circadian regulation by CYP monooxygenases affects the synthesis of secondary metabolites in various organisms. Here, light exposure led to the downregulation of CYPs as circadian reporters but did not directly affect the *F. velutipes* fruiting body color. Moreover, levels of some metabolic-pathway-related genes, such as *Fvpal1* and pyridoxal-dependent decarboxylase, were significantly upregulated under blue light. Upregulation of the expression of *Fvpal1*, an enzyme involved in phenylpropanoid metabolism, suggests its potential roles in pigment biosynthesis and fruiting body color changes in *F. velutipes* under blue light. Here, a pyridoxal-dependent decarboxylase gene associated with AAA metabolism and secondary metabolite biosynthesis was identified, further emphasizing the intricate relationship between light exposure and metabolic processes in mushroom development. These findings not only contribute to our understanding of fungal biology but also reveal new molecular markers for further exploration of light-mediated regulatory pathways in fruiting body forming fungi.

## Figures and Tables

**Table 1:** Significantly enriched differentially expressed genes (DEGs) in blue- and green-light-treated *Flammulina velutipes*.

Gene ID	Treated light	log2FC	FDR ^1^	Pfam Database *(p* < 0.001)		Family
				ID	Description	
TRINITY_DN1447_c0_g1_i1	Blue	−2.0926	0.0000	PF00067.26	Cytochrome P450	CYP620
	Green	−1.5818	0.0002			
TRINITY_DN6930_c0_g1_i17	Blue	−1.3246	0.0000	PF00067.26	Cytochrome P450	CYP53
	Green	−1.2260	0.0015			
TRINITY_DN5360_c0_g1_i15	Blue	−1.5705	0.0000	PF00067.26	Cytochrome P450	CYP620
	Green	−1.4023	0.0212			
TRINITY_DN5015_c0_g3_i1	Blue	−2.2012	0.0001	-	-	
	Green	−2.2610	0.0158			

^1^ False discovery rate (<0.05).

**Table 2:** Hub genes in the *Fvpal1* protein–protein interaction (PPI) network identified using cytoHubba in Cytoscape software.

Gene ID	Closeness	Degree	Pfam Database		KEGG ^1^ Database	
			ID	Description	ID	Description
TRINITY_DN3704_c0_g1_i1	10	10	PF00155.25	Aminotransferase classes I and II	K00817	Histidinol-phosphate aminotransferase [EC:2.6.1.9]
TRINITY_DN5155_c0_g1_i16	10	10	PF00155.25		K14455	Aspartate aminotransferase, mitochondrial [EC:2.6.1.1]
TRINITY_DN5724_c0_g1_i3	10	10	PF00282.23	Pyridoxal-dependent decarboxylase conserved domain	-	-
TRINITY_DN631_c0_g1_i10	9.5	9	PF00155.25	Aminotransferase classes I and II	K14454	Aspartate aminotransferase, mitochondrial [EC:2.6.1.1]
TRINITY_DN631_c0_g1_i5	9.5	9	PF00155.25		K14454	
TRINITY_DN2176_c0_g1_i14	8	6	PF00155.25		K00838	Aromatic amino acid aminotransferase I/2-aminoadipate transaminase [EC:2.6.1.57 2.6.1.39 2.6.1.27 2.6.1.5]
TRINITY_DN2176_c0_g1_i24	8	6	PF00155.25		K00838	
TRINITY_DN9451_c0_g1_i2	8	6	PF00800.22	Prephenate dehydratase	-	
TRINITY_DN9451_c0_g1_i39	8	6	PF00800.22			
TRINITY_DN9451_c0_g1_i45	8	6	PF00800.22			

^1^ Kyoto Encyclopedia of Genes and Genomes.

## Data Availability

Data are contained within the article and Supplementary Materials.

## Supplementary Materials

The following supporting information can be downloaded at https://www.mdpi.com/article/10.3390/jof10060372/s1. Table S1: List of primers used in this study. Table S2: Differentially expressed genes (DEGs) in *Flammulina velutipes* treated with different light-emitting diodes (LEDs). Table S3: Enrichment of 168 downregulated DEGs in *Flammulina velutipes* treated with blue and green lights. Table S4: List of enriched genes in *Flammulina velutipes* treated with blue and green lights. Table S5: List of hub genes identified in this study.
